# Remove, Refine, Reduce: Cell Death in Biological Systems

**DOI:** 10.3390/ijms24087028

**Published:** 2023-04-10

**Authors:** Marcus Krüger

**Affiliations:** Environmental Cell Biology Group, Department of Microgravity and Translational Regenerative Medicine, Otto von Guericke University, 39106 Magdeburg, Germany; marcus.krueger@med.ovgu.de

## 1. Cell Death as a Component of Development and Homeostasis

Cell death is an important biological phenomenon. In this process, which is strictly regulated by the organism, old or damaged cells are destroyed for replacement by new cells. Additionally, cells that are infested with microbes are eliminated to fight infections. Like other cellular processes (e.g., cell proliferation or cell differentiation), cell death is a mechanism determined by the cell, either voluntarily or accidentally.

**Figure 1:**
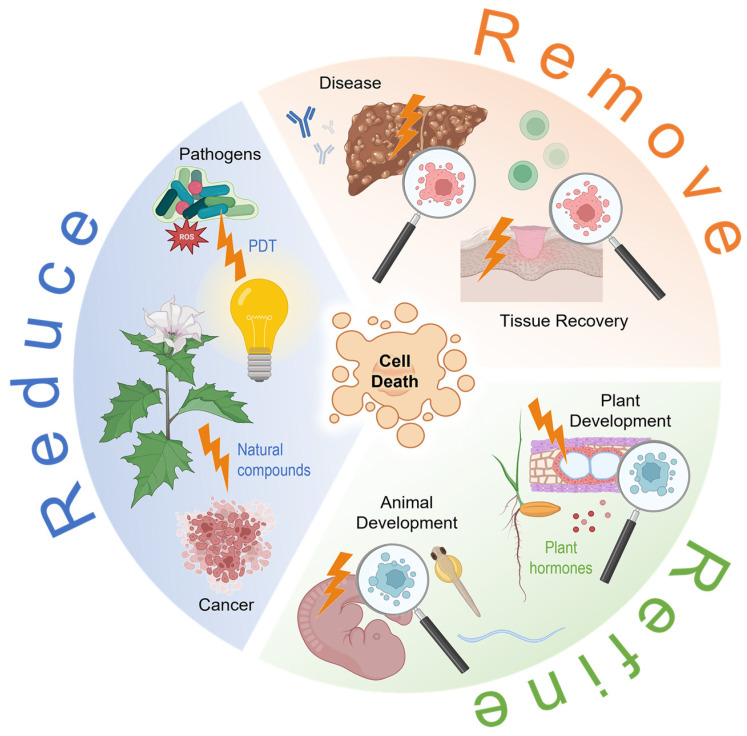
Illustrated overview of the various functions and occurrences of cell death in organisms, with an emphasis on the topics covered in this Special Issue. PDT, photodynamic therapy; ROS, reactive oxygen species. The figure was created using elements from BioRender.com (accessed on 30 March 2023).

In simplified terms, a cell can die in two ways. One such way is apoptosis, also known as programmed cell death or cell suicide, a physiological form of cell death and a process of active cell death under the control of genes. The other is necrosis, an uncontrolled form of cell death that occurs in response to ischemia and other injury. Studies have shown that there are a number of distinct, but interrelated, processes that all end in the death of a cell [1,2,3]. More detailed insights into the different types of cell death are highlighted in the second volume of this Special Issue [4]. This volume focuses on the study of cell death in various cells, tissues, organisms, and during physiological processes (Figure 1).

## 2. Remove

Cell death is an important process in the elimination of unwanted cells. For example, white blood cells are removed after an infection has been cleared or when cells are infected with viruses or microbes.

### 2.1. Cell Death in Disease

The European Union’s Horizon *Research and Innovation* Magazine [5] states that “when the body machinery that kills off hundreds of millions of cells a day fails, inflammation and sickness are often not far behind”. Currently, it is well established that cell death plays a fundamental role in tissue homeostasis. Abnormal regulation of this process is associated with a variety of human diseases, including developmental disorders, neurodegeneration (exacerbated cell death), immunological disorders, and cancer (insufficient cell death). Hepatocyte cell death is also a crucial event in the progression of liver disease, as it triggers inflammation that eventually leads to fibrosis. Regulated cell death is central to the severity and outcome of liver injury. For this Special Issue, Shojaie et al. [6] summarized the known signaling cascades in the various cell death pathways, and their impact on liver disease. Meanwhile, Yin et al. [7] demonstrated that hepatocyte-derived Igκ plays a protective role in concanavalin A-induced liver injury in mice, and that Igκ appears to be essential for hepatocyte survival. Knockdown of Igκ promoted hepatocyte apoptosis by accelerating activation of the mitochondrial death pathway and cleavage of caspase-3 in vitro.

### 2.2. Cell Death Caused by Exogenous Effects

Polyhexamethylene guanidine phosphate (PHMG-p) is a water-soluble disinfectant found in hospitals and residential facilities. It is also used in shampoos, wipes, cleaning products, detergents, paints, and swimming pools, due to its broad-spectrum antimicrobial properties [8,9]. Epidemiological and toxicological studies have shown a strong association between this chemical and pulmonary fibrosis [10]. The studies by Jeong et al. [11] suggest that PHMG-p rapidly localizes the endoplasmic reticulum (ER) and triggers ER stress-mediated apoptosis, which is the first step in PHMG-p-induced pulmonary fibrosis.

As summarized by Prasad et al. [12], exposure to a space environment has been shown to affect apoptosis in several cell types. On the one hand, the reduced gravity disturbs the balance between cell architecture and the external force, leading to changes at the cellular and subcellular levels (e.g., cytoskeleton, signal transduction, membrane permeability, etc.). On the other hand, cosmic rays can cause complex DNA damage, along with damage of other cells, due to high linear energy transfer.

## 3. Refine

Cell death is an important process when cells are no longer needed, for example, at certain stages of development. In addition, some structures in the body, such as the outer layer of the skin, consist of dead cells.

### 3.1. Cell Death during Development

Nicotinamide adenine dinucleotide phosphate (NADPH) production by glucose-6-phosphate dehydrogenase (G6PD) is critical for reductive biosynthesis and redox homeostasis in cells [13]. G6PD is required for embryonic development in animals, as severe G6PD deficiency was shown to be lethal in mice and nematodes [14,15]. The study by Yang et al. [16], using G6PD-deficient *Caenorhabditis elegans*, indicated that increased lipid peroxidation promotes germ-cell death and impairs embryogenesis in *C. elegans*.

In the regulation of plant development and programmed cell death, the plant hormone, jasmonate, is an important signaling molecule [17], promoting the degradation of jasmonate ZIM (zinc-finger inflorescence meristem) domain (JAZ) proteins to reverse the repression of several transcription factors that execute jasmonate responses [18]. Feng et al. [19] identified the JAZ protein, OsJAZ13, in *Oryza sativa* as a negative regulator of jasmonate signaling in rice, which appears to play an important role in cell death in plants. It suppresses the expression of *MYC2* and *ERF1*, gradually inducing the expression of defense-related genes, and modulating the balance between defense and growth under unfavorable environmental conditions.

### 3.2. Cell Death in Tissue Recovery

Cell death also plays a critical role in restoring and maintaining skin homeostasis. It supports recovery from acute injury and infection, and regulates barrier function and immunity [20]. Tumor necrosis factor (TNF) is a pleiotropic cytokine which is important for retaining tissue homeostasis by controlling both cell death and inflammation. Several checkpoints determine whether TNF leads to cell death or survival [21]. Key molecules in cell death signaling, such as cFLIP (cellular FLICE-like inhibitory protein), have also been shown to be essential for maintaining skin homeostasis, since the loss of any of these molecules resulted in severe inflammatory skin disease. Feoktostova et al. [22] have now developed and analyzed a double-knockout mouse model, in which TNF is completely absent and cFLIP is exclusively absent in keratinocytes. Using this new genetic mouse model, they were able to confirm that the inflammatory skin disease resulting from the epidermal deletion of cFLIP is strongly, but not exclusively, dependent on TNF.

## 4. Reduce

Cell death is an important process when cells are damaged, e.g., by radiation or toxins. Induction of this process is particularly useful when unwanted cells, such as tumor or pathogen cells, are to be combated.

### 4.1. Induced Cell Death to Fight Cancer

Lipocalin-2 (LCN2) expression is markedly altered in many cancers [23]. The glycoprotein appears to promote tumorigenesis by increasing invasion, metastasis and proliferation, and decreasing apoptosis. Santiago-Sánchez et al. [24] summarized key findings on the expression, biological role, and regulation of LCN2, as well as the proteins with which LCN2 interacts in cancer. They also discussed approaches to targeting cancer with LCN2 that are currently under investigation, including the use of RNA interference, antibodies, and gene editing.

It has been previously reported that the combination of the choline-binding domain of the amidase N-acetylmuramoyl-L-alanine-D-amino acid oxidase (CLytA-DAAO) and D-alanine induces cell death in several pancreatic and colorectal carcinomas, and glioblastoma cell lines [25]. Fuentes-Baile et al. [26] have now found that CLytA-DAAO induces apoptotic and necrosis-like cell death, depending on the cellular context. The team of authors identified several cell-death-induced mechanisms operating in different tumor cell lines. This ability to adapt to the environment of each cancer type and trigger cell death by different mechanisms gives CLytA-DAAO a broad range of potential cell targets and opens the possibility of using the enzymes in many types of cancer cells.

Medicinal plants contain numerous compounds that can be used to kill cancer cells. In recent years, it has been found that it is not only apoptosis that plays a role, but that some substances also promote non-canonical cell death [27]. The sweet bitter leaf, *Vernonia calvoana*, contains high concentrations of flavonoids, which can act as antioxidants and prevent cell damage in living organisms. The study by Mbemi et al. [28] showed that one of the crude extract fractions of *V. calvoana* was able to inhibit cell proliferation, induce DNA damage, and arrest the cell cycle at the S-phase checkpoint in OVAR-3 human ovarian cancer cells. Lim et al. [29] found that pygenic acid A, a natural compound from *Prunella vulgaris*, not only induced apoptosis, but also sensitized the two metastatic triple-negative breast cancer cell lines, MDA-MB-231 (human) and 4T1 (mouse), to anoikis. Pygenic acid A downregulated survival-promoting proteins, including cIAP1, cIAP2, and survivin, resulting in cell death of both attached and suspended cells.

### 4.2. Induced Cell Death to Combat Pathogens

Bacteria are also able to control cell death under various stress conditions, such as high temperatures, amino acid deficiency, or antibiotic treatment [30]. Photodynamic inactivation (often referred to as antimicrobial photodynamic therapy, aPDT) is an emerging method for controlling bacteria when the use of antibiotics or other methods is not possible or desirable. Spacecraft and space stations contain a diverse population of microorganisms [31,32], some of which exhibit altered metabolism [33]. Antibiotics should be used sparingly in the space environment, as their use as a preventive measure in a simulated microgravity environment could be counterproductive and is likely to lead to persistent antibiotic resistance [34]. Buchovec et al. [35] have summarized ways to use antibacterial photodynamic inactivation based on natural photosensitizers, to combat bacterial biofilms in spacecrafts.

## 5. Conclusions

Cell death may sound unwelcome, but it is essential for the survival of every human being and serves a specific purpose in the biology of all multicellular organisms. The mechanisms of cell death are evolutionarily conserved, and the elements are also found in unicellular organisms. The molecular pathways of cell death (or, conversely, cell survival) are part of a comprehensive signaling network. Cell death is an essential part of the body’s recycling of cellular building blocks, and is, in fact, a precondition for life. Every second, one million cells die in our body. This means that in one day, approximately 1.2 kg of cells die in a healthy human. However, that should not worry us. The disruption of cell death mechanisms often leads to developmental abnormalities, and factors that trigger cell death can directly contribute to the development of many diseases, including cancer, Alzheimer’s disease, and tissue damage. In plants, programmed cell death also plays an important role, e.g., in the formation of the sclerenchyma and xylem for water and mineral transport. In a way, death seems to be an essential component of life. We would like to thank all the authors who contributed to this Special Issue and added further pieces to the puzzle of understanding cell death as a complex and critical process in living organisms.
